# Electron microscopic imaging and NanoSIMS investigation on physiological responses of *Aspergillus niger* under Pb(II) and Cd(II) stress

**DOI:** 10.3389/fbioe.2022.1096384

**Published:** 2023-01-12

**Authors:** Shang Pan, Zhaoyan Li, Jiayi Wang, Xuefei Li, Lingzi Meng, Yunhui Chen, Mu Su, Zhen Li

**Affiliations:** ^1^ College of Agro-grassland Sciences, Nanjing Agricultural University, Nanjing, China; ^2^ College of Resources and Environmental Sciences, Nanjing Agricultural University, Nanjing, China; ^3^ Jiangsu Key Laboratory for Organic Waste Utilization, Nanjing Agricultural University, Nanjing, China; ^4^ State Key Laboratory of Palaeobiology and Stratigraphy, Nanjing Institute of Geology and Palaeontology, Nanjing, China

**Keywords:** lead, cadmium, *Aspergillus niger*, electron microscopy, NanoSIMS, GWB simulation

## Abstract

In the bioremediation process, coexistence of lead (Pb) and cadmium causes complex toxicity, resulting in the difficulty of bioremediation. This study investigated the physiological responses and bioaccumulation mechanisms of the typical filamentous fungus *Aspergillus niger* under the coexistence of Pb and Cd. Four treatments were set up, i.e., control, sole Pb, sole Cd, and coexistence of Pb and Cd. The morphology of *A. niger* were observed by scanning electron microscopy (SEM) and transmission electron microscopy (TEM), respectively. Then, nano-scale secondary ion mass spectrometry (NanoSIMS) was applied to accurately investigate the distribution of heavy metals in the fungal cells under the coexistence of Pb and Cd. Finally, the metallogenic process and mineral types were simulated by Geochemist’s Workbench (GWB). The electron microscopic and NanoSIMS imaging showed that Pb and Cd were accumulated in both the extracellular and intracellular regions of the *A. niger* cells. In particular, the accumulated Pb content was ten times higher than that of Cd. However, Cd showed stronger toxicity than Pb to *A. niger*. Compared with the control treatment, Cd stress resulted in a two-fold increase of cell diameter and more extracellular substances, whereas the cell diameter increased nearly four times in the coexistence treatment. Moreover, the bioaccumulation of Pb was more intense than that of Cd during competitive sorption. The GWB simulation confirmed that Pb^2+^ can form multiple minerals (e.g., PbC_2_O_4_, PbHPO_4_, and Pb_3_(PO_4_)_2_, *etc.*), which significantly weakened its toxicity on the cell surface. This study elucidated the morphological characteristics of *A. niger* and competitive bioaccumulation under the coexistence of Pb and Cd, which would facilitate the application of microorganisms to the bioremediation of coexisted metals.

## 1 Introduction

Heavy metal pollution caused by anthropogenic activities is increasing (Jarup, 2003; Hou, 2021). The coexistence of heavy metals in sewage and solid wastes derived from mining, smelting, and electroplating industries usually causes compound pollution (Song et al., 2022). Lead (Pb) and cadmium (Cd) are the two most common heavy metals (Yang et al., 2018). According to the national communique of soil pollution survey by the Ministry of Environmental Protection of China, the over-limit rates of Pb and Cd were 1.5% and 7.0%, respectively (MEP of China, 2014). Thus, the coexistence of Pb and Cd is one of the most common combined pollutions (Gao et al., 2010; Chen et al., 2015).

*Aspergillus niger* is a representative phosphate-solubilizing fungus in soil (Khan et al., 2014). It could produce abundant organic acids and extracellular degradative enzymes to accelerate the release of phosphate (Tian et al., 2021). Thus, *A. niger* has been widely applied to bioremediation (Tian et al., 2020; Yang et al., 2020). *Aspergillus niger* had more stable heritability and a strictly stronger acid-producing capacity than bacteria and many other fungi (Sharma et al., 2013; Yu et al., 2021). The oxalic acid (H2C2O4) secreted by *A. niger* could efficiently precipitate heavy metal cations (Yakout, 2014). In addition, heavy metals could be accumulated in both intracellular and extracellular regions of fungal cells (Kapoor and Viraraghavan, 1997; Qiu et al., 2021; Geng et al., 2022). Thus, the filamentous fungus *A. niger* has been considered as an ideal strain for heavy metal bioremediation (Ahluwalia and Goyal, 2007; Ren et al., 2009; Zegzouti et al., 2020). However, previous studies mostly focused on remediation of a single heavy metal (Gola et al., 2016; Tian et al., 2019; Okolie et al., 2020). Therefore, lacking knowledge of bioaccumulation under the coexistence of Pb and Cd impeded the application of microorganisms in the remediation.

The sorption capacity of *A. niger* to Pb was usually higher than that of Cd due to their different affinity to negative charges on cell surface (Amini & Younesi, 2009; Okolie et al., 2020). *Aspergillus niger* had higher tolerance concentrations of Pb than Cd, i.e., >1,500 mg/L for Pb and only 100 mg/L for Cd (Tian et al., 2019; Okolie et al., 2020). Moreover, Pb-oxalate was easier to be precipitated than Cd-oxalate, as the solubility product constant (*Ksp*) of Pb-oxalate is nearly three orders of magnitude less than Cd-oxalate (Pb oxalate: *Ksp* = 2.74 × 10^−11^; Cd oxalate: *Ksp* = 1.42 × 10^−8^) (Benitez and Dubois, 1999; Johansson et al., 2008). Therefore, the responses of *A. niger* to Pb and Cd should be correlated to a series of factors under the coexistence system.

Scanning electron microscopy (SEM) and transmission electron microscopy (TEM) were suitable for observing the surface morphology and internal structure of microorganisms, respectively (Jiang et al., 2021; Su et al., 2021). In the interaction between microorganisms and heavy metals, the metallogenesis and mineral crystal structure were observed by SEM (Bhattacharya et al., 2018; Chen et al., 2019; Tian et al., 2019; Xu et al., 2021). Significant changes in the sizes of microbial cells have been observed under metal stimulation based on SEM imaging (Gola et al., 2016; Sharma et al., 2017; Jiang et al., 2020). In addition, TEM could identify intracellular and extracellular adsorption of heavy metals based on its resolution up to nanometre scale (Zhu et al., 2017). Furthermore, the fine observation by TEM elucidated a new cell wall formation under Pb stress (Tian et al., 2019).

Nano-secondary ion mass spectrometry (NanoSIMS) owns high sensitivity when investigating microchemistry (Guerquin-Kern et al., 2005). Recently, the potential of NanoSIMS as a new tool in the study of bio-interface has been demonstrated (Yu et al., 2020). The high sensitivity, high lateral resolution (50 nm for Cs^+^ primary ion beam source), and high mass resolution (∼4,000X) for secondary ions qualify the NanoSIMS as a powerful tool for investigating elemental composition (e.g., C, N, P, and halogen elements) on microbial samples (Popa et al., 2007). However, NanoSIMS technology is rarely applied to the studies of microbial responses to heavy metals.

In this study, we investigated the morphological responses and metallogenic mechanisms of *A. niger* to the coexistence of Pb and Cd. SEM and TEM were used to elucidate morphology characteristics and internal structures of *A. niger* cells. Then, NanoSIMS was applied to identify the distribution of cell composition elements and heavy metals. Finally, based on Geochemist’s Workbench (GWB), the mineralization of Pb and Cd cations was simulated, which would provide a theoretical understanding of bioremediation.

## 2 Materials and methods

### 2.1 Fungal strain and incubation

*Aspergillus niger* strain information can be referred to our previous study (Qiu et al., 2021). The fungus accession number in China General Microbiological Cultural Collection Center (CGMCC) is No. 11544. *Aspergillus. niger* was cultured in potato dextrose agar (PDA) medium at 28 °C for 5 days. After spore formation, the medium was drenched with sterile water. The spores were scraped carefully from the plate surface with a fine brush. Then, the suspension was filtered through a three-layer sterile cheesecloth to eliminate mycelial fragments. The concentration of spores was measured by haemacytometer. The initial count of spores was 10^7^ cfu mL^−1^.

### 2.2 Experimental design

Four treatments were performed, i.e., CK (no metal addition), TPb (sole Pb addition), TCd (sole Cd addition), and TPbCd (addition of Pb and Cd). The concentrations of Pb and Cd addition were both .893 mmol/L. Three replicates were set for each treatment. The solid Pb(NO_3_)_2_ powder (Xilong Scientifc Ltd.) and Cd(NO_3_)_2_ powder (98% cadmium nitrate tetrahydrate, Sigma Aldrich Inc.) were added to 100 mL potato dextrose broth (PDB) medium. After sterilizing the medium, 1 mL spore suspensions were added to the medium for incubation. The initial pH value of the inoculation system was set as 6.5. All the treatments were incubated at 28°C for 5 days under 180 rpm shaking.

### 2.3 Experimental instruments and analytical methods

After the incubation, the precipitates and supernatant were separated by centrifugation (2,504 rcf, 10 min). The precipitates were dried at 65°C for 24 h for subsequent analyses.

#### 2.3.1 SEM analysis

The samples were fixed by 2.5% glutaraldehyde for 4 h. After the samples were rinsed with .1 M sodium phosphate buffer (pH = 7.4), ethanol of 30%, 50%, 70%, 85%, 90%, and 100% was used for dehydration of the precipitates. Finally, isoamyl alcohol was applied to dry the precipitates in a freeze-dryer for 48 h. The samples were pasted on the platform with conductive adhesive for SEM analysis. The image acquisition was tested by Carl Zeiss SUPRATM 55 system. Gold particles by Gressington 108 Autosputter coated the samples to improve electrical conductivity and prevent thermal damage. Semi-quantitative analysis was performed by Oxford Aztec X-Max 150 energy dispersive X-ray spectrometer (EDS).

#### 2.3.2 TEM analysis

The processing of the samples for TEM analysis can refer to our previous study (Tian et al., 2019). The precipitate was pre-fixated with electron microscopy fixative (G1102, Servicebio, Wuhan, China) and fixed again by osmic acid. The samples were prepared as ultrathin sections (60–80 nm thickness). The field-emission transmission electron microscope was performed by FEI Tecnai G2 F20S-TWIN system equipped with AZtec X-Max 80T energy dispersive spectrometer (EDS).

#### 2.3.3 NanoSIMS analysis

The precipitates collected from the TPbCd treatment were analyzed by NanoSIMS. The sample preparation processes were similar to the process for preparation of TEM samples. After embedding, the sample was sectioned with 400 nm thick slices. The element observations were performed with a NanoSIMS 50 (Cameca, Courbevoie, France). A Cs + primary ion beam was used to continuously bombard microbial cells on the sample surface. Then, the secondary ions were sputtered and liberation from the upper surface. These secondary ions were sorted based on their energy in the electrostatic sector before being dispersed in a mass spectrometer according to their mass/charge ratios. By acquiring a series of spatially referenced spectra, maps of ^16^O^−^、^12^C^14^N^−^ (characterize nitrogen (N)), ^208^Pb^16^O^−^ and ^114^Cd^16^O^−^ were produced for the atomic mass.

#### 2.3.4 GWB modeling

Geochemist’s Workbench (GWB 11, Aqueous Solutions LLC.) was applied to simulate mineralization of the metals. Under the Titration mode, using React module to simulate ion concentration changes with pH value in the system. The concentrations of Pb^2+^ and Cd^2+^ in the system were set based on the experimental design. The maximum concentration of H_2_PO_4_^−^ was set to 10 mmol/L (Qiu et al., 2021). The phase diagram of the dominant minerals was drawn by Act2 module. The mineralization of Pb and Cd were subsequently simulated when reaching an equilibrium state at each site of the system.

## 3 Results

### 3.1 SEM and EDS analyses

**FIGURE 1:**
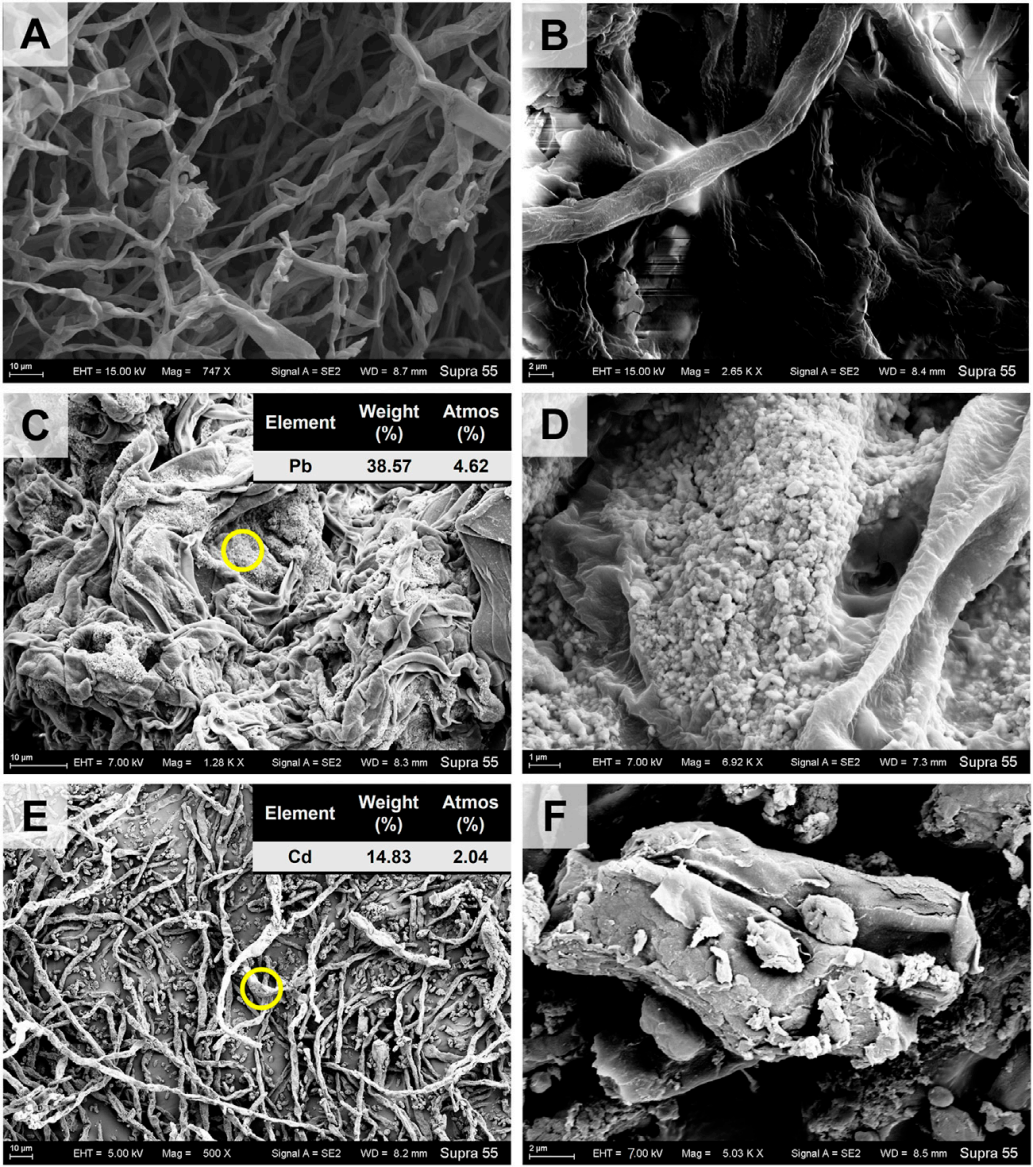
The SEM images of *Aspergillus niger* after 5 days incubation in the CK **(A, B)**, TPb **(C, D)**, and TCd **(E, F)** treatments. The representative particles on the mycelial surface in images C and E were selected for EDS analysis.

In the CK treatment, the typical diameter of *A. niger* hypha was ∼2 μm (Figures 1A,B). In the TPb treatment, the hypha has a typical diameter of ∼3 μm (Figures 1C,D). The value increased to 5–6 μm in the TCd treatment (Figures 1E,F). Moreover, the hyphae were tightly interwoven under Pb stress, but loosely arranged under Cd stress.

**FIGURE 2:**
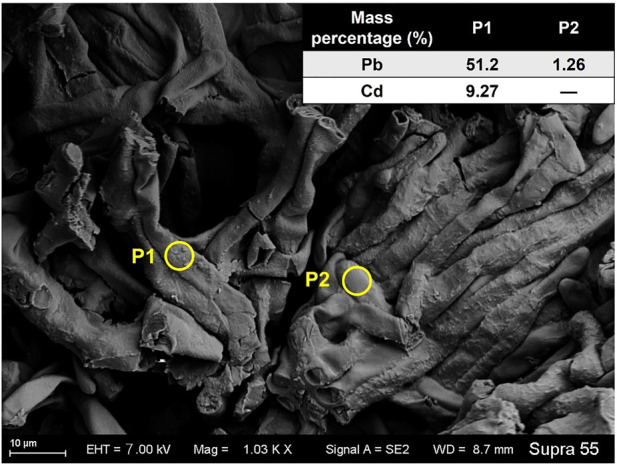
The SEM image of *Aspergillus niger* after 5 days incubation in the TPbCd treatment. The representative spots of P1 and P2 were selected for EDS analysis.

The mycelia showed rough surface with the enrichment of particles. In the TPb treatment, the particle diameter varied between .1–.5 μm (Figure 1D). In contrast, the particle diameter in the TCd treatment was larger, i.e., 1–2 μm (Figure 1F). The representative particles on the mycelial surface were selected for EDS analysis. In the TPb and TCd treatments, the weight percentage of Pb and Cd accounted for 38.57% and 14.83%, respectively (Figures 1C,E). In the TPbCd treatment, the mycelia were arranged tightly and orderly. The diameter of hypha was ∼5–6 μm, which was much larger than those in the other treatments (Figure 2). Meanwhile, the Pb and Cd weight percentage at P1 was 51.2% and 9.27%, respectively. The content of Pb was nearly five times higher than Cd. The Pb content at P2 was 1.26 wt%, yet Cd was under the detection line (Figure 2).

### 3.2 TEM and EDS analyses

**FIGURE 3:**
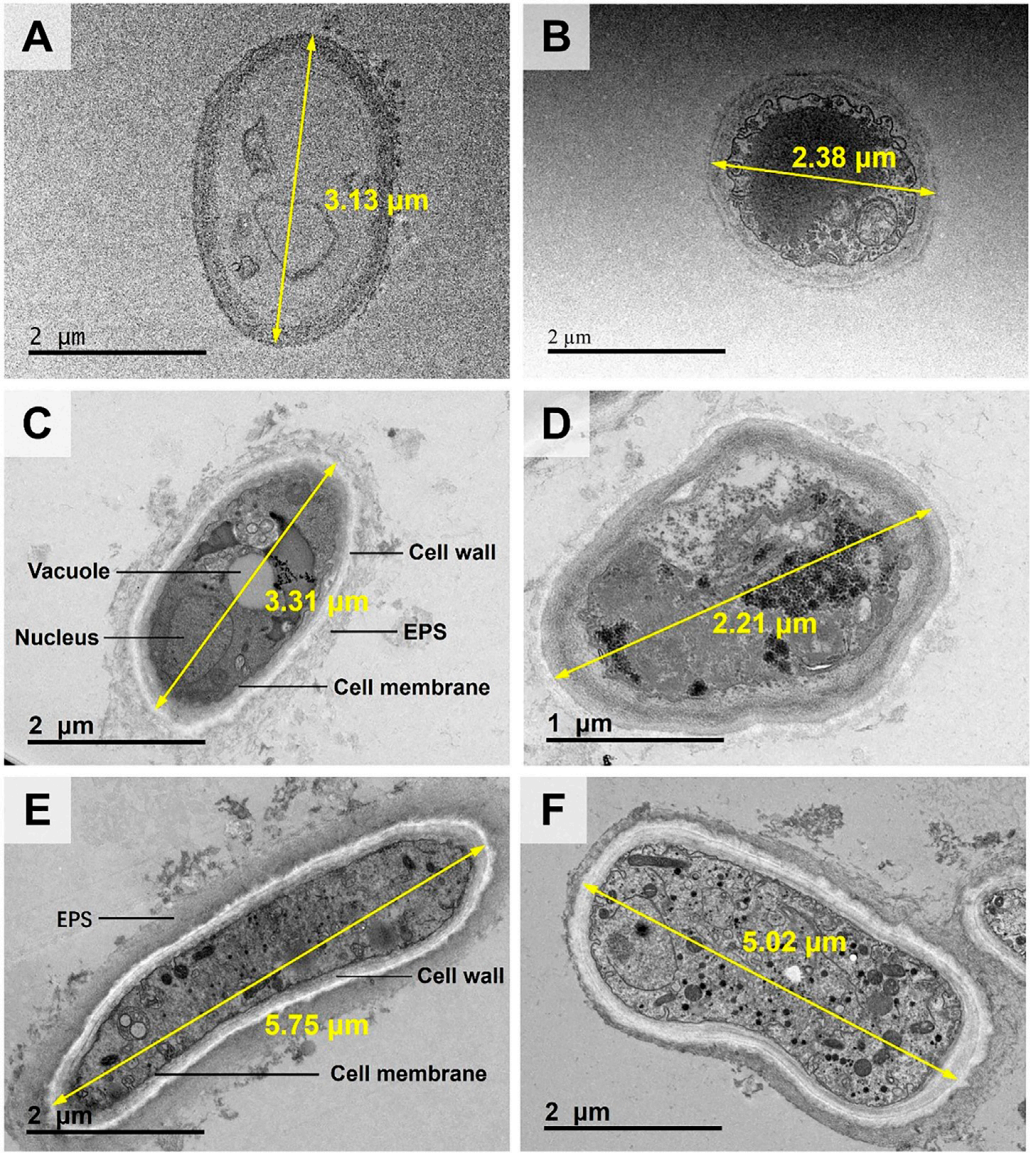
The TEM images of morphological changes of *Aspergillus niger* after 5 days incubation in the CK **(A, B)**, TPb **(C, D)**, and TCd **(E, F)** treatments. Cell diameters of representative cells were shown.

In the CK treatment, the cell diameter was about 2–3 μm (Figures 3A,B). There was almost no extracellular substances. The cell wall thickness was around .1 μm. Moreover, no evident black particles were observed (see Figures 3A,B). In the TPb treatment, the cell size or the cell wall thickness showed no significant change (Figures 3C,D). However, the abundance of extracellular substances was increased, which were attached loosely to the cell walls. The particles were enriched near the vacuoles (Figures 3C,D). In the TCd treatment, the cell diameters were increased to 5–6 μm, and the cell wall thickness was increased to .3 μm. Meanwhile, the extracellular substances were secreted to form a dense layer outside the cells. Moreover, the particles were not only distributed in the intracellular region, but also adsorbed on the extracellular substance surfaces (Figures 3E,F).

**FIGURE 4:**
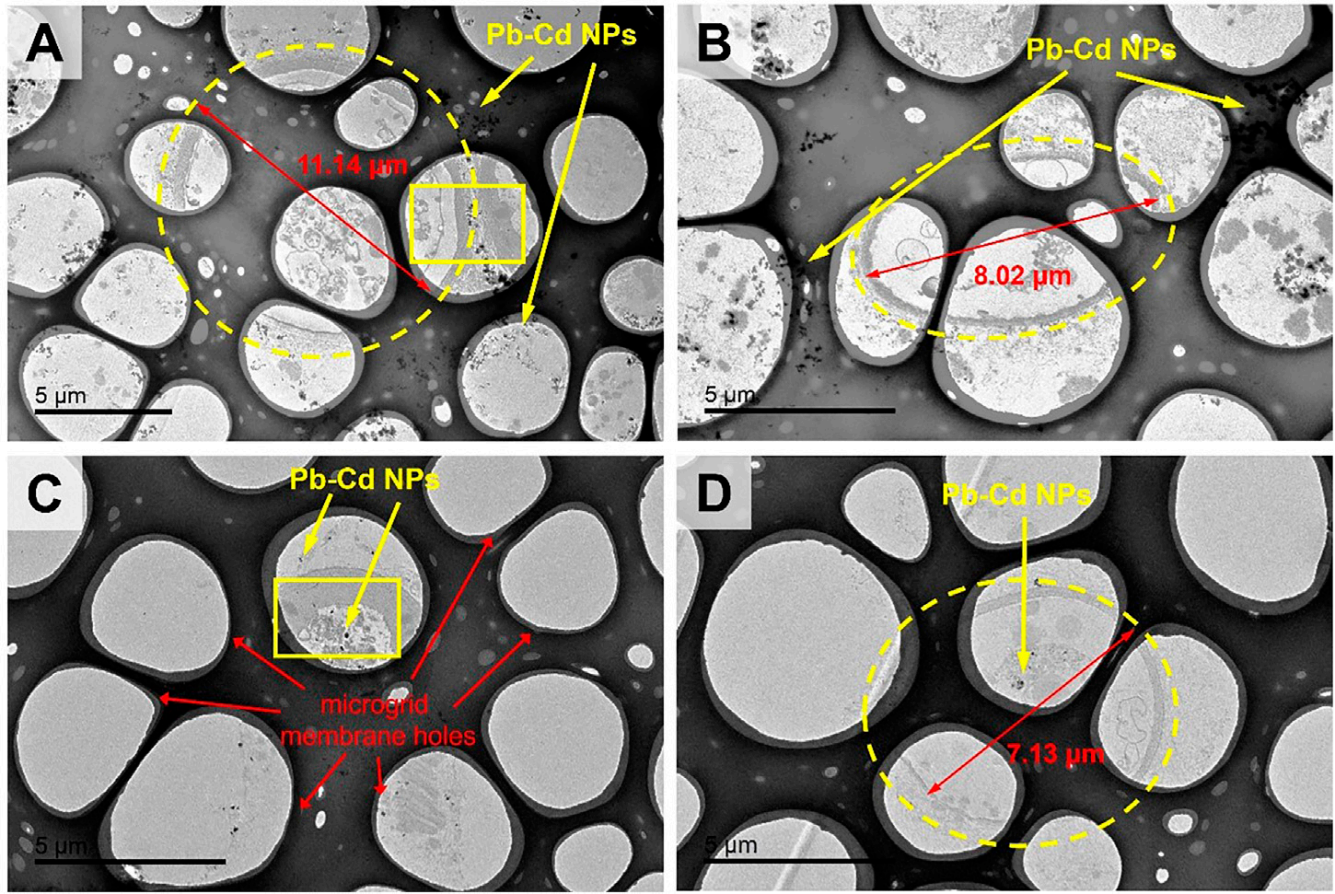
The TEM images of morphological changes of *Aspergillus niger* after 5 days incubation in the TPbCd treatment. Cell diameters of several representative cells were shown. Representative Pb and Cd nano-particles (NPs) were shown.

In the TPbCd treatment, the cell diameter was enlarged to 7–11 µm under TEM, which was about twice of that in the TCd treatment and four times of that in the CK treatment (Figure 4). Moreover, the particles were enriched in both the extracellular and intracellular regions of the cells (Figure 4). It should be noted that the circles with a diameter of ∼5 µm were the microgrid membrane holes. The cells of *A. niger* were marked by dotted circles (Figure 4).

**FIGURE 5:**
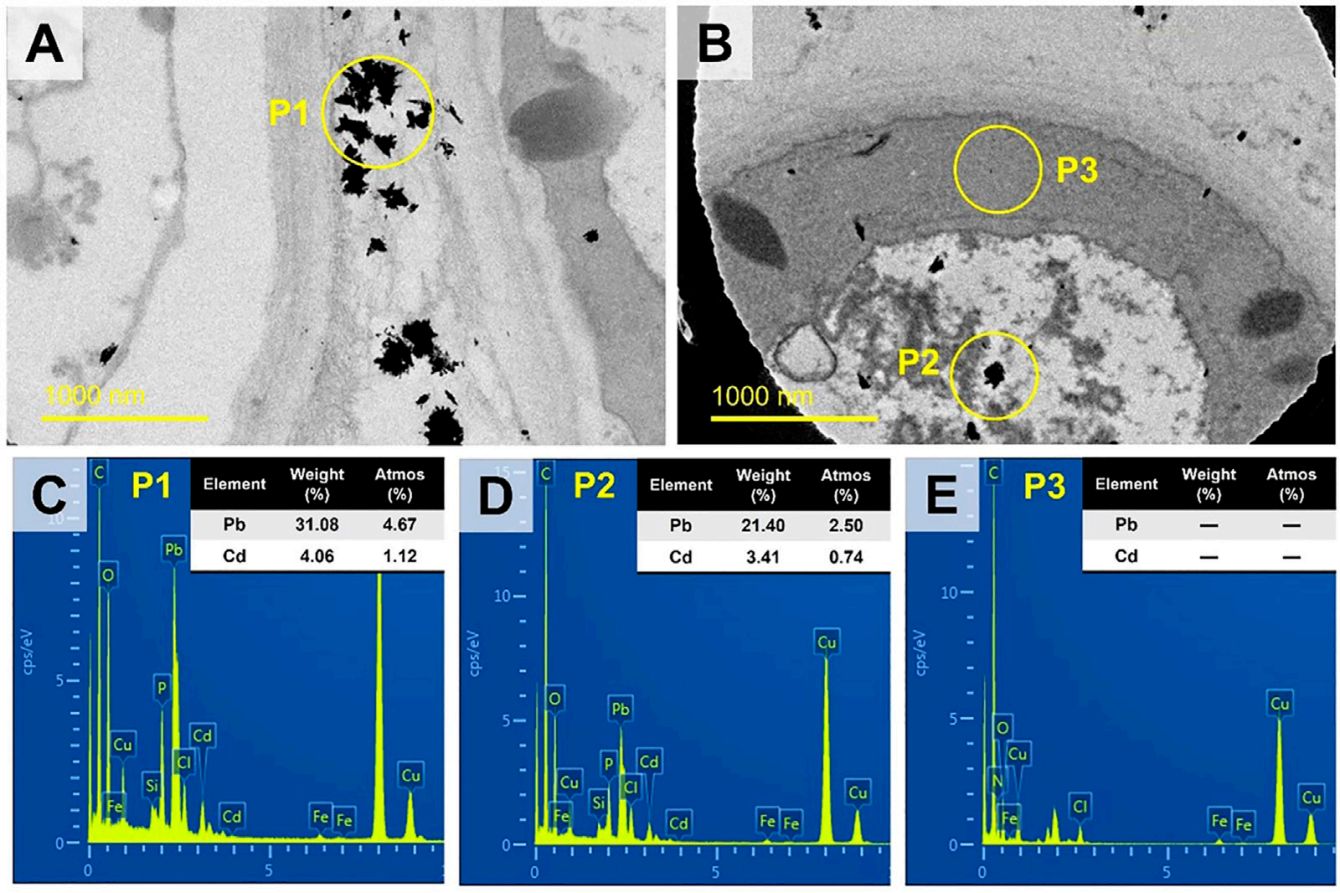
The TEM images of the rectangular regions in Figures 4A image **(A)** and 4C image **(B)** at high-resolution (slight offset might occur). Three representative spots (P1, P2, P3) were selected for EDS analysis as shown in images **(C–E) **respectively.

The representative micro-regions (marked as rectangular in Figure 4C) were selected for high-resolution observation. It showed that the particles aggregated in the extracellular region, while dispersedly distributed in the intracellular region (Figures 5A,B). The weight percentage of Pb in P1 and P2 was 31.08% and 21.40%, while that of Cd was as low as 4.06% and 3.41%, respectively. No signal of Pb or Cd was detected in P3 (Figures 5C–E).

### 3.3 NanoSIMS analysis

**FIGURE 6:**
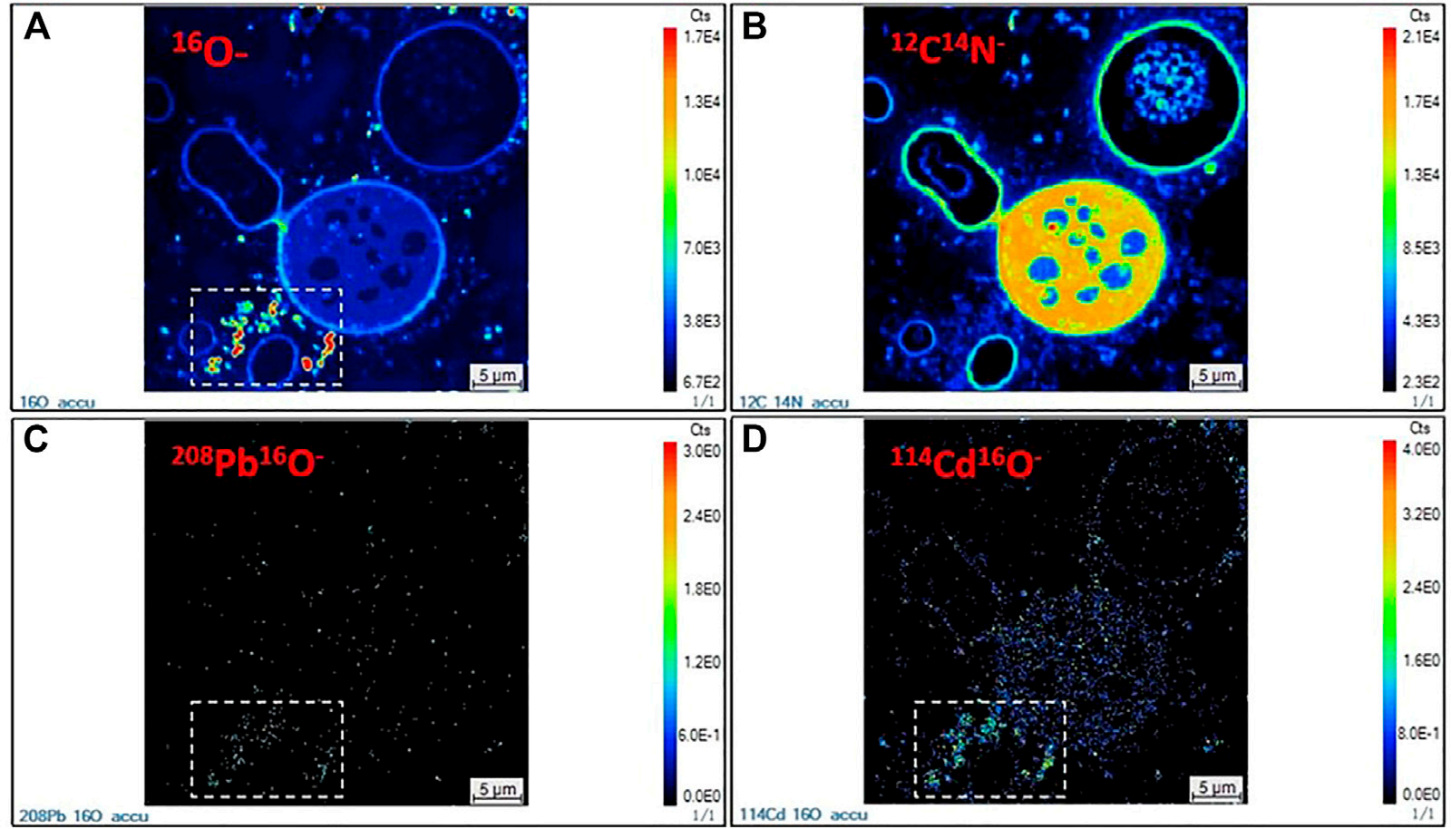
NanoSIMS images of *Aspergillus niger* cells in the TPbCd treatment. **(A)** ^16^O^−^ secondary ion image; **(B)** ^12^C^14^N^−^ secondary ion image; **(C)** ^208^Pb^16^O^−^ secondary ion image; **(D)** ^114^Cd^16^O^−^ secondary ion image.

The spatial distribution of the secondary ions ^16^O^−^, ^12^C^14^N^−^, ^208^Pb^16^O^−^, and ^114^Cd^16^O^−^ under the TPbCd treatment were displayed in Figure 6. The intense ^16^O^−^ signals were indicated in the dashed rectangular area (see Figure 6A). In contrast, the strong ^12^C^14^N^−^ signals appeared in the intracellular region (Figure 6B). The ^12^C^14^N^−^ was used to characterize the contour and position of cells as N has been considered as an indicator of biogenic matters (Romer et al., 2006). Moreover, several weak ^12^C^14^N^−^ signal circles were observed, which might be attributed to the dead cells undergoing/after cytoplasm decomposition. In addition, the enrichment of both ^208^Pb^16^O^−^ and ^114^Cd^16^O^−^ were higher in the extracellular region than that in the intracellular region (Figures 6C,D).

### 3.4 GWB simulation

**FIGURE 7:**
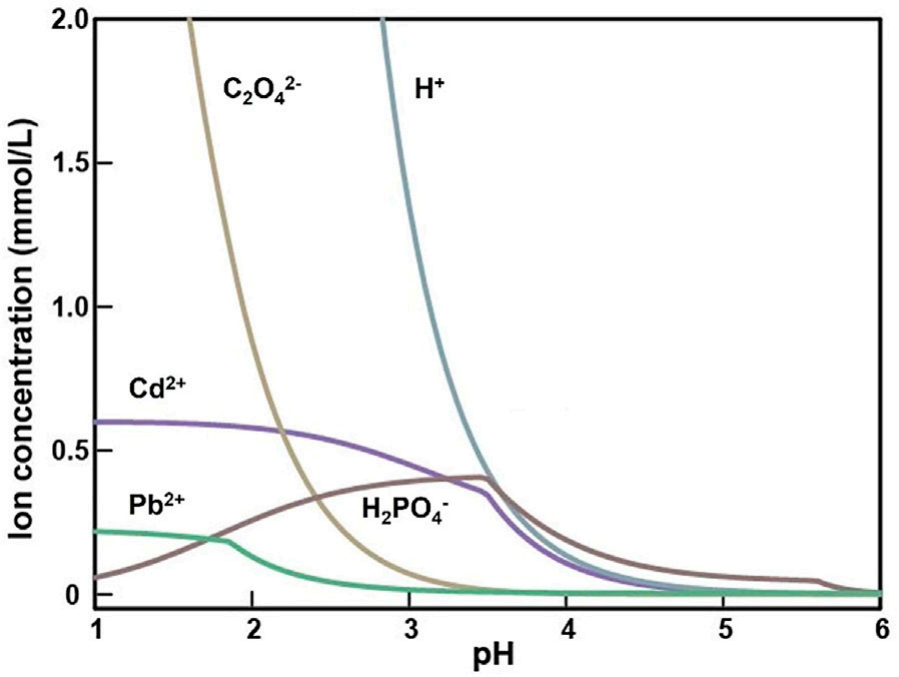
The concentration curve of the main ion species varying with pH value after 5 days incubation in the TPbCd treatment.

The geochemical modeling under the TPbCd treatment showed that the concentrations of Pb^2+^ and Cd^2+^ were decreasing along with the decline of H^+^ and C_2_O_4_^2−^ concentrations (Figure 7). In addition, the Pb^2+^ concentration was always lower than that of Cd^2+^, which indicated that Pb^2+^ was easier to form mineralized precipitation (with occurrence of C_2_O_4_^2−^) than Cd^2+^ (Figure 7).

**FIGURE 8:**
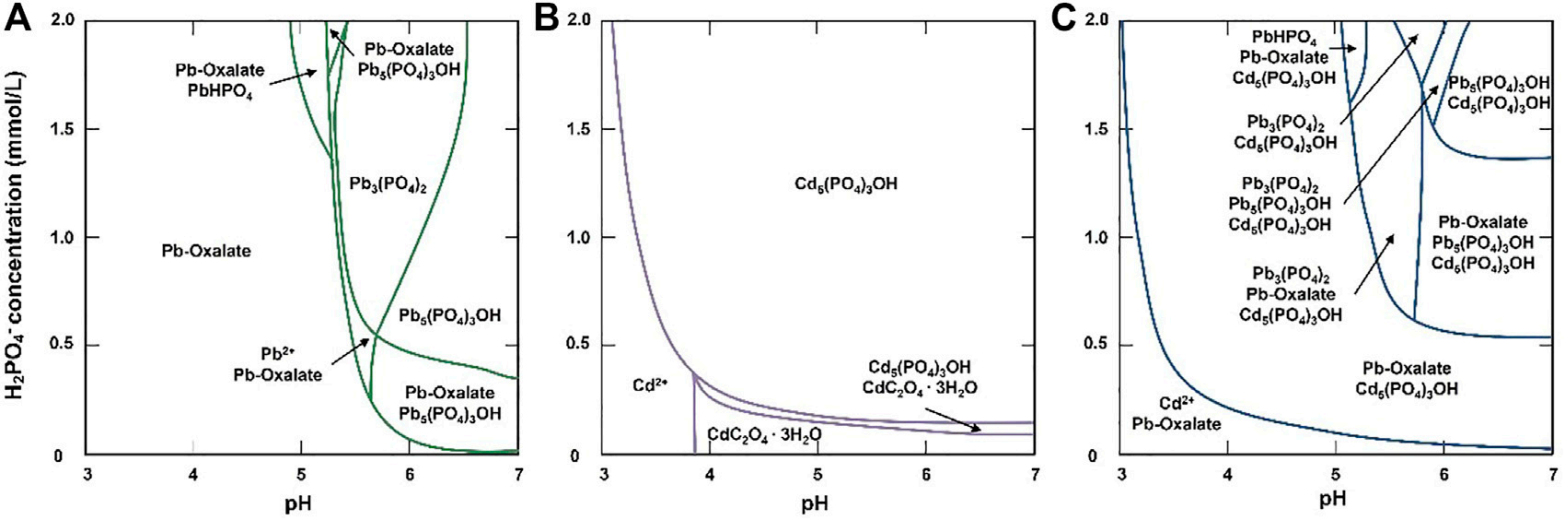
Diagrams of Pb and Cd phase with the changes of pH and H_2_PO_4_^−^ concentrations in the TPb treatment **(A)**, TCd treatment **(B)**, and TPbCd treatment **(C)**.

The phase diagrams revealed the mineralization processes of Pb and Cd (Figure 8). In the TPb treatment, the mineral was Pb-oxalate when pH < 5.2, while the mineral types increased to Pb_3_(PO_4_)_2_, PbHPO_4_, and Pb_5_(PO_4_)_3_OH when pH > 5.2 (Figure 8A). In the TCd treatment, most Cd existed as free cations when pH < 3. When pH < 3.8 and H_2_PO_4_^−^>.4 mmol/L, the system was dominated by Cd_5_(PO_4_)_3_OH. When pH > 3.8 and H_2_PO_4_^−^<.4 mmol/L, oxalate minerals dominate the mineralization (Figure 8B).

In the TPbCd treatment, Pb presented as Pb-oxalate when pH value and H_2_PO_4_^−^ concentrations were relatively low (Figure 8C). Compared with the TCd treatment, Cd-oxalate was not formed when pH > 3.8 and H_2_PO_4_^−^ < .4 mmol/L (Figures 8B,C). Only when pH and H_2_PO_4_^−^ concentrations continue to increased, Cd^2+^ cations were mineralized to Cd_5_(PO_4_)_3_OH. In addition, Pb induced a variety of minerals, such as Pb_3_(PO_4_)_2_, PbHPO_4_, and Pb_5_(PO_4_)_3_OH (Figure 8C).

## 4 Discussion

In this study, *A. niger* showed distinct responses to Pb and Cd stresses. This was consistent with the conclusion that Pb concentrations <1,000 mg/L could promote biological activity (Sayer et al., 1999). Therefore, the secretion of extracellular substances would subsequently be promoted (Figure 3). However, the tolerance of A. *niger* to Cd was much weaker due to its high toxicity and migration (Wu et al., 2016). The biomass of *A. niger* was significantly lower under Cd stress than the CK and Pb treatments (Wang, et al., 2017; Qiu, et al., 2021). The dead cell lysis would also release intracellular organic matters (Li, et al., 2021), which were adsorbed around the living cells to isolate the contact between the cells and heavy metals. In addition, the extracellular substances and cell debris had similar functional groups (Li, et al., 2012), which were mainly composed of proteins, polysaccharides, lipids, and humic acids (Lin et al., 2014). Their negatively charged functional groups could adsorb heavy metal cations (Comte et al., 2008; Dang et al., 2018). Therefore, the abundance of extracellular organic substances are able to form a protective layer of *A. niger* to immobilize metals.

Microbes can adapt to environmental changes by regulating their morphology (Guan et al., 2020). This study revealed the phenomenon of the enlargement of the cross-section of hyphae under the coexistence of Pb and Cd (Figure 3). Moreover, appropriate Pb^2+^ in the coexistence system could significantly enhance microbial activities by promoting the tricarboxylic acid cycle of *A. niger* (Qiu et al., 2021). This response to heavy metals was also observed in *A. niger* sporangia which were increased by 50% (Xu et al., 2021). When *A. niger* was exposed to heavy metals, it preferred to promote the surface area by expanding the cell volume. The larger cell surface provided more active sites for adsorbing more heavy metal ions (Smyth, 1989). This mechanism was also consistent with the study regarding the resistance of bacteria to Cd toxicity (Keene et al., 2008).

The NanoSIMS mapping showed that the bioaccumulation of Pb and Cd was more intense in the extracellular than intracellular region. *Aspergillus niger* could secrete a variety of low-molecular-weight organic acids (LMWOAs) (Strobel, 2001; Li et al., 2016). Oxalic acid was the most abundant LMWOAs (Yakout, 2014). Compared with other LMWOAs, oxalic acid had a higher acidity constant (p*Ka*1 = 1.25; p*Ka*2 = 4.27), which facilitated the formation of oxalate precipitation to reduce metal toxicity (Green and Clausen, 2003). In addition, Pb had higher competitive accumulation than Cd in the coexistence system. The competitive accumulation of Pb and Cd also existed in the bioremediation by bacteria. For example, a study of *Pseudomonas putida* showed that Pb^2+^ had almost the same sorption sites as Cd^2+^ on the cell surface (Du et al., 2016). Moreover, the bioaccumulation efficiency of *Exiguobacterium* sp. to Pb was also higher than that of Cd (Park and Chon, 2016).

The GWB simulation showed that Pb^2+^ and Cd^2+^ competed for oxalate species (C_2_O_4_^−^) in the coexistence system (Figure 7). Pb^2+^ was preferred to generate oxalate minerals due to that Pb-oxalate usually has a lower *Ksp* value than Cd-oxalate (Benitez and Dubois, 1999). Furthermore, Pb^2+^ could form a variety of mineral species (e.g., Pb oxalate (PbC_2_O_4_), PbHPO_4_, and Pb_3_(PO_4_)_2_). However, Cd^2+^ cations were commonly mineralized as Cd_5_(PO_4_)_3_OH. Additionally, it was attributed to the stronger affinity between Pb and amino acid residues, which induces the *Ksp* of Pb-containing compounds lower than that of Cd (Table 1). Therefore, in the coexistence of Pb and Cd, Pb^2+^ was more easily mineralized. The mineralization finally immobilized and detoxified the cations.

**TABLE 1:** Solubility product constants (*Ksp*) of typical Pb and Cd compounds.

Functional groups	Chemical formula	*Ksp*	References
Pb			
Carboxyl groups	-COOH	2.74 × 10^−11^	Lee et al. (1999)
Phosphate groups	-H_2_PO_4_	8.0 × 10^−43^	Martinez et al. (2004)
Hydroxyl	-OH	1.2 × 10^−15^	Frost and Williams (2004)
Carbonate	-CO_3_	7.4 × 10^−14^	Yao et al. (2013)
Chromate	-CrO_4_	2.8 × 10^−13^	Zheng et al. (2017)
Sulfate radical	-SO_4_	1.6 × 10^−8^	DeSantis et al. (2018)
Cd			
Carboxyl groups	-COOH	1.42 × 10^−8^	Wang et al. (2017)
Phosphate groups	-H_2_PO_4_	2.53 × 10^−33^	Dmitrevskii et al. (2008)
Hydroxyl	-OH	5.27 × 10^−15^	Canepari et al. (1998)
Carbonate	-CO_3_	1.0 × 10^−12^	Remacle et al. (1992)

## 5 Conclusion

This study identified the physiological responses and metallogenetic mechanisms of *A. niger* to Pb and Cd stress. Our findings confirmed that the filamentous fungus *A. niger* had multiple pathways to effectively adsorb heavy metal ions, e.g., producing LMWOAS, secreting extracellular substances, and enlarging the cell surface area. Therefore, *A. niger* shows evident advantages in the bioremediation of heavy metals. In the coexistence system, Pb had preferential bioaccumulation than Cd, which allowed that most Pb cations could be mineralized and detoxified. This study sheds a light on the remediation of the coexistence of metals by functional fungi.

## Data Availability

The raw data supporting the conclusions of this article will be made available by the authors, without undue reservation.
